# Comprehensive Insight from Phthalates Occurrence: From Health Outcomes to Emerging Analytical Approaches

**DOI:** 10.3390/toxics9070157

**Published:** 2021-07-01

**Authors:** Catarina Luís, Manuel Algarra, José S. Câmara, Rosa Perestrelo

**Affiliations:** 1 CQM-Centro de Química da Madeira, Campus da Penteada, Universidade da Madeira, 9020-105 Funchal, Portugal; cgsluis@staff.uma.pt (C.L.); jsc@staff.uma.pt (J.S.C.); 2 Faculdade de Ciências da Vida, Unidade de Ciências Médicas, Universidade da Madeira, Campus Universitário da Penteada, 9020-105 Funchal, Portugal; 3 Department of Inorganic Chemistry, Faculty of Science, Campus de Teatinos s/n, University of Málaga, 29071 Malaga, Spain; malgarra67@gmail.com; 4 Departamento de Química, Faculdade de Ciências e Engenharia, Campus da Penteada, Universidade da Madeira, 9020-105 Funchal, Portugal

**Keywords:** phthalates, health risk, extraction techniques, analytical approaches

## Abstract

Phthalates are a group of chemicals used in a multitude of important industrial products (e.g., medical devices, children’s toys, and food packages), mainly as plasticizers to improve mechanical properties such as flexibility, transparency, durability, and longevity of polyvinyl chloride (PVC). The wide occurrence of phthalates in many consumer products, including foods (e.g., bottled water, soft drinks, wine, milk, and meat) brings that most people are exposed to phthalates every day, which raises some concerns. Adverse health outcomes from phthalates exposure have been associated with endocrine disruption, deformities in the human reproductive system, increased risk of preterm birth, carcinogen exposure, among others. Apprehension related to the health risks and ubiquitous incidence of phthalates in foods inspires the development of reliable analytical approaches that allow their detection and quantification at trace levels. The purpose of the current review is to provide information related to the presence of phthalates in the food chain, highlighting the health risks associated with their exposure. Moreover, an overview of emerging extraction procedures and high-resolution analytical approaches for a comprehensive quantification of phthalates is presented.

## 1. Introduction

Phthalates, generally known as phthalate esters (PAEs, Supplementary Material), are a family of chemicals used industrially in a wide variety of consumer products, primarily as plasticizers (e.g., substances added to plastics to increase their flexibility, longevity, durability, and transparency), is durable, flexible polyvinyl chloride (PVC) applications and polyvinyl acetate, largely for the construction, automotive, wire and cable sectors, in addition to non-PVC applications such as rubber products, sealants, adhesives, and coatings. Generally, they are organized into two main distinct groups: higher-molecular weight (HMW) phthalates (chemical backbone with 7–13 carbon atoms), and lower-molecular weight (LMW) phthalates (chemical backbone with 3–6 carbon atoms), with differentiated applications, legal requirements, and toxicological properties. While the LMW phthalates, such as butyl benzyl phthalate (BBP), di-n-butyl phthalate (DBP), and diethyl phthalate (DEP), are mainly used as solvents in different consumer and personal care products, HMW phthalates including di-isononyl phthalate (DiNP), and di-2-ethylhexyl phthalate (DEHP), are primarily used as plasticizers to soften PVC products. The structures, common uses, and health effects of phthalates commonly monitored in foods and packaging materials are summarized in Table 1.

Besides being easily released into the environment, they are rapidly biodegraded and photodegraded, leading to a lower persistence.

**Figure 1:**
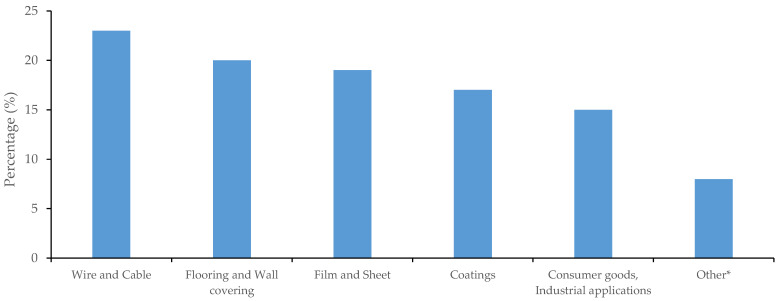
Average distribution of plasticizers use in Europe (2020). Other*: surface coatings, rubber compounds, medical applications, and elastomers (Source: 2020 IHS and European Plasticizers estimates).

The most common exposure routes of phthalates are: (i) personal care products (e.g., shampoos, deodorants, soaps, perfumes, nail polish, and body lotions); (ii) food contact plastics (e.g., bottled water and food transporting containers) [7]; (iii) sucking or chewing soft plastic/vinyl products (e.g., plasticizers used in children’s products) [5]; (iv) medical devices (e.g., catheters and blood bags). In addition, phthalates can be found in wall coverings, coated textiles, sports equipment, footwear, electrical cables, and house flooring. Figure 1 summarizes the distribution of the application of plasticizers in Europe in 2020.

From the described potential routes, food and beverage constitute, undoubtedly, the most important source of human exposure to phthalates [8,9]. Particularly, bottled water, due to its high and regular consumption, has drawn considerable attention. Besides the polyethylene terephthalate (PET), the most common polymer used in bottled water packaging is reported to be free from phthalates, as few studies have shown the presence of phthalates in bottled water packed in PET containers [10,11,12]. Dairy products, infant formula, meat, baked goods, fats and oils, and fast foods are major contributors to dietary phthalates exposure. Therefore, the monitoring exposure of chemicals from packaging materials into foods has become a fundamental part of ensuring food safety and protecting human health.

**Figure 2:**
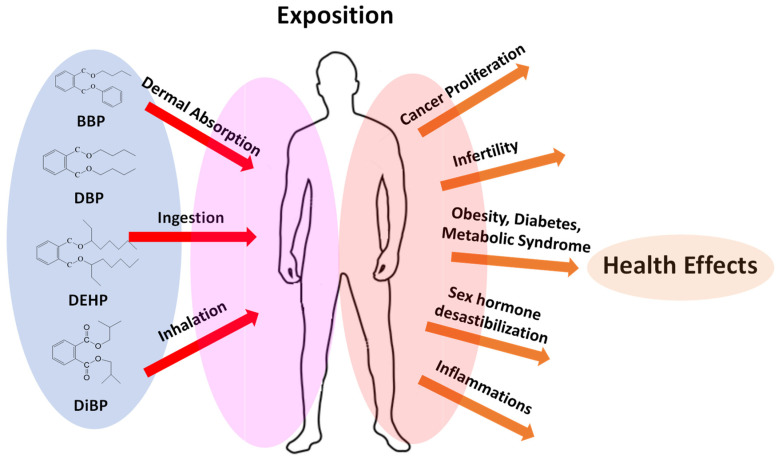
Human exposition to phthalates and their health effects.

The phthalates are naturally released into the environment through their production, use, and/or disposal, and therefore can be absorbed by the human body by different routes (skin absorption, inhalation, and ingestion), as seen in Figure 2 [3]. Few studies have examined the health effects of phthalates on humans. In lab animals, phthalate exposure has been found to be associated with numerous reproductive health and developmental problems such as the early onset of puberty, interference with the male reproductive tract development, and with the natural functioning of hormonal systems.

According to Bennett et al. [13], exposure from the prenatal stage of childhood to some phthalates discloses an unacceptably high risk of future developing neurologic disorders (e.g., autism and intellectual disabilities). In addition, the exposition to phthalates might cause a reduction in testosterone levels in adolescent males and a decreased sperm count in adult males. When absorbed as androgen blocking chemicals and weak endocrine disruptors, phthalates can suppress the hormones involved in male sexual development and can either mimic or block male and female hormones. In Europe [14], most phthalates are banned in plastic food contact materials for fatty food, including dairy products and infant food.

In the following sections, we provide descriptive information related to the presence of phthalates in the food chain, highlighting the health risks associated with their exposure. An overview of the emerging extraction procedures and analytical approaches for a comprehensive quantification of phthalates is also discussed. For this purpose, the keywords “phthalates”, “PAEs”, “environmental”, “food”, “healthy risks”, “extraction technique”, and “analytical platforms” were researched in Pubmed, Scopus, Web of Science, and Google Scholar over the period of 2015 to 2021. It should be pointed out that other references were included outside of the established period due to their relevance to this review.

## 2. Phthalates Background

As previously mentioned, PAEs are a class of synthetic chemicals mainly obtained from petroleum and added to an enormous number of everyday products. PAEs, linear and branched, are added to improve the properties of plastic materials (e.g., softness, flexibility, transparency, durability, and longevity) [15]. Lately, the cosmetic industry has added them to fragrances, perfumes, and especially lotions, nail polish, hair spray, and soap, as a vehicle for these preparations, providing the feeling that their effects last longer [16]. However, such applicability raises a current concern in the European Union due to the possibility of adverse effects when they are added to toys and their handling by young children due to their tendency to put them in the mouth [17]. Bekö et al. [18] analyzed the total daily intakes (TDI) of DEP, DnBP, DiBP, BBzP, and DEHP based on metabolites levels in the urine of 431 Danish children between 3 and 6 years of age. The results obtained showed that DEHP had the highest TDI (median: 4.42 µg/d/kg-bw) and BBzP the lowest (median: 0.49 µg/d/kg-bw). For DEP, DnBP, and DiBP, exposures to air and dust in the indoor environment accounted for approximately 100%, 15%, and 50% of the total intake, respectively, with dermal absorption from the gas-phase being the major exposure pathway. More than 90% of the TDI of BBzP and DEHP result from other sources such as indoor air and dust [18]. Based on these, health care organizations have begun investigating their risks to human health.

### 2.1. Human Exposure Routes

Due to the widespread use of these types of compounds and their immense applications, there is a potential risk, for both children and adults, of being exposed to phthalates. Phthalates are relatively released from the products into the environment due to the weak chemical bond between phthalates and other chemicals due to their dipolar interactions [19].

Maternal diet and food preparation practices, such as maternal prenatal high-fat milk consumption was associated with higher benzyl butyl (BBz) and DEHP [20]. The presence of these endocrine disruptors, DBP, DEP, dioctyl phthalate (DOP), was found in vegetable cans, baby bottles, microwaveable containers [5,21], and the well-known plastic beverage bottles [7]. As mentioned, the simple contact of a child’s mouth and saliva with their toys can contract PAEs and fastly converted them into respective metabolites [22]. The phthalate plasticizer DEP is illegally used in clouding agents and used in foods and beverages [23].

Non-dietary exposure is another source, where they can be emitted from materials into the air and easily partitioned into the indoor and outdoor environment. Different phthalates have been detected, such as DEHP, diisobutyl phthalate (DiBP), and di-n-butyl phthalate (DnBP) in soil dust [24]. The drawback with phthalates is that once they are already in the indoor environment, their elimination becomes difficult. It must be taken into account that people that have spent a lot of time indoors, for several years [25], leading to health effects [26,27]. Phthalates have been detected in a variety of medical devices, such as intravenous tubing, umbilical artery catheters, blood bags and infusion tubing, enteral nutrition feeding bags, nasogastric tubes, among others. Tubing is normally used in cardiopulmonary bypass procedures, in extracorporeal membrane oxygenation, during hemodialysis, and during peritoneal dialysis. Their flexibility can make medical devices easier to use and less likely to cause damage to tissues, and they are also more comfortable for patients [28,29,30].

### 2.2. Healthy Risks

There are numerous studies where it has been shown that phthalates can alter the endocrine system and induce a plethora of effects such as carcinogens, teratogens, and mutagens [31,32].

**Figure 3:**
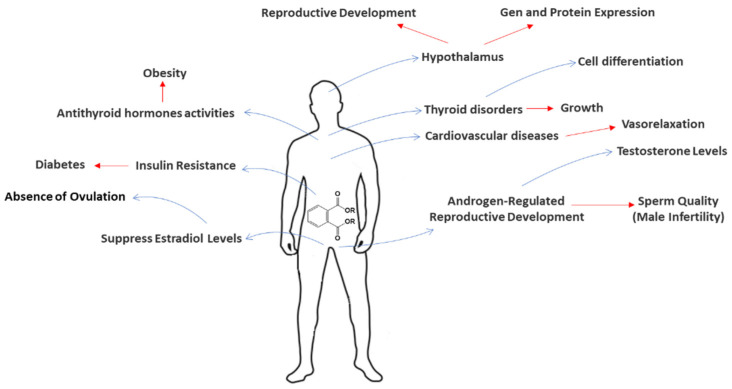
Main hormonal action of high levels of PAEs associated with humans.

Many phthalates, even at a low concentration, are known endocrine disruptors (Figure 3) that have an influence on the development of organisms and their reproductive system [33]. For instance, PAEs have been recognized to induce changes in oxidative stress, disturb sex hormone balances, which can decrease fertility and increase the rate of reproductive defects and malformations [33,34,35,36]. Interference with testosterone activity, especially early in life, can have irreversible effects on male reproduction [37]. Evidence of the existence of infertility in male animals has been found in terms of a drastic decrease in sperm and some other malformations related to the reproductive system [28].

Phthalate exposure in humans has been linked to metabolic changes, such as increasing obesity problems (metabolic syndrome) [38] and the inherent chronic illnesses associated with diabetic episodes [6,39]. Other studies related to the toxicity of phthalates monoesters have been demonstrated possible alterations in the gene expression of antioxidant enzymes, thyroid endocrine, and balance of sex hormone disrupting their effects [33,40,41].

Some other studies have concluded there is a relationship between phthalate accumulation (DEHP, BBP, and DBP) and breast cancer [42]. Furthermore, it was suspected that the interference with the cell cycle is related to genes and, therefore, to cancer proliferation. Other studies revealed that exposure to phthalates increases tumor activity in terms of activation of different signaling processes [43]. Taking all these into account, our review provides strong evidence that the presence of phthalates plays an important role in the proliferation of different cancer stem cells by interference in the associated signaling processes [2,42,43].

Several mechanisms have been proposed to explain the increase in blood pressure in pregnancy due to exposure to phthalates, namely an increase in oxidative stress, a decrease in serum thyroxin, and an increase in inflammatory cytokines, which could promote gestational hypertension or preeclampsia development [3,44,45,46,47]. Su et al. [48] assessed the relationship between phthalate exposure and atherosclerosis in young populations. The data obtained showed that DEHP and DBP have a significant correlation with carotid intima-media thickness, an indicator of atherosclerosis development. Regarding human in vitro studies, the data obtained demonstrated that MEHP leads to apoptosis and may be promoted by increased autophagosomes mediated by ROS in a mitochondrial-dependent manner in human endothelial cells [3]. Sicińska [1], also in vitro, assessed the effects of DBP, BBP, and their respective metabolites upon the induction of apoptosis in human peripheral blood mononuclear cells. The data obtained showed an increase in calcium levels and caspases activity and a decrease in transmembrane mitochondrial potential, which indicates significant pro-apoptotic alterations [1]. Another correlation between PAEs and the development of some cardiometabolic risks, including hypertension, heart disease, stroke, and atherosclerosis, has been extensively discussed by Mariana and Cairrao [3].

### 2.3. Phthalates Regulation

Due to the generalized exposure of phthalates, as mentioned above, in a great diversity of products, both at a domestic and industrial level, the different international health agencies had to regulate the levels of these chemical contaminants.

In the United States, the Environmental Protection Agency (EPA) regulated the presence of eight compounds, namely DBP, DiBP, BBP, di-n-pentyl phthalate (DnPP), DEHP, di-n-octyl phthalate (DnOP), DiNP, and diisodecyl phthalate (DiDP) in consumers [37,38], restricting the daily intake of all of them to 20 μg/g of body weight [49], and also regulated their presence as excipients in products, namely DBP [50]. In 2019, the European Food Safety Agency (EFSA) established TDI of 0.05 mg/kg for DBP, BzBP, DEHP, and DiNP [2,51]. The European Union (EU) established the Directive 2011/65/EU ‘RoHS Recast’, which was amended recently to include the presence of phthalates as BBP, DBP, DEHP, DiDP, and DiNP used as additives or polymer production aids in domestic devices and toys, in order to control their use [52]. This was recently modified with the Directive (EU) 2015/863, where the presence of four phthalates (BBP, DBP, DEHP, DiDP, and DiNP) was limited to be used as additives or coadjutants for the production of polymers. They are listed as restricted substances in Annex II of the Directive 2011/65/EU (RoHS 2) since they are used in domestic devices or toys. Regarding the EU, member states intend to apply their adopted provisions starting on 22 July 2019. The highlights of this Directive state that the maximum concentration values tolerated by weight in homogeneous materials is 0.1% [53]. In China, DMP, DEP, and DnOP have been listed as priority pollutants by the China National Environmental Monitoring Center [54]. BBP is controlled in Canada according to the Canada Consumer Products Safety Act: Phthalates Regulations. These regulations restrict the usage of phthalates, including BBP, in soft vinyl children’s toys and child care articles to not more than 1000 mg/kg of DEHP, DBP, or BBP [55]. Australia and Japan have an important directive for phthalate regulations [2,56]. Moreover, the USA, Australia, New Zealand, and Japan have established a DEHP maximum level in drinking water at 6, 9, 10, and 100 µg/L, respectively [57]. Many other regulations and guidelines related to soil, water, sediment, and sludge have been compiled by Net et al. [58].

## 3. Occurrence of Phthalates

### 3.1. Environmental

PAEs are a group of chemicals that are widely used as plasticizers [59,60,61]. In this sense, they became common environmental contaminants since they tend to migrate into the environment during the disposal of the PAE-containing product [62]. As a result, there is a risk of exposure of phthalates in humans, leading to their accumulation in several matrices such as soil, seawater, sediments, sludge, surface waters, among others [60,62,63,64,65,66,67]. Thus, the monitoring of their levels is imperative due to the possible implications for human health. These compounds are usually extracted from the matrices using several extraction procedures being the most common the liquid-liquid extraction (LLE) [68], ultrasound-assisted extraction (UAE) [61], solid-phase microextraction (SPME) [60,69], followed by gas chromatography coupled with mass spectrometry (GC-MS). GC-MS analysis is still the analytical approach of choice in many cases for target and non-target analysis of these target compounds [70]. Table 2 summarizes the recent investigations related to the determination of PAEs in environmental and in food samples. Moreover, Ning et al. [67] used accelerated solvent extraction (ASE) to determine the levels of phthalates (DMP, DEP, DBP, and DEHP) in mine tailings. The obtained limit of detection (LOD) and limit of quantitation (LOQ) values were in the range of 1.2–2.0 µg/kg and 3.0–4.6 µg/kg, respectively, while recoveries ranged from 71% to 115%. Zhang et al. [64] analyzed the seamount area of the Tropical Western Pacific Ocean, and the concentrations ranged from 12.13 ng/L to 60.69 ng/L. The recoveries obtained were ranged between 93% to 97%. Among the 14 PAEs detected, DBP, DEHP, and DiBP were also dominant in the surface seawater samples. Jebara and collaborators [63] monitored the presence of PAEs in seawater, sediment, seagrass, and fish from several sites along the Tunisian coast. The levels ranged from 5 to 763 µg/g, with the higher levels being obtained in fish and sediments, while seagrass presenting the lower levels. Another study with sediments was performed by Lee et al. [62] that determined the concentrations of PAEs in sediment samples collected along the Korean coast. They verified that the higher amounts were obtained for harbors, suggesting that they were contaminated hotspots. The average levels ranged from 24.3 to 3700 ng/g, while for non-phthalate plasticizers varied from 0.32 to 92.2 ng/g. Furthermore, Hu et al. [71] also analyzed PAEs sediment samples collected from several China bays, and the detected PAEs were in the range of 654 to 2603 ng/g. With regard to soil samples, Rodrígues-Ramos at al. [72] used several nanomaterials as an alternative method to extract the PAEs from soil samples and, by using the 1,3,5-benzenetricarboxylate metal-organic framework, the best results were obtained. The LODs obtained varied from 0.14 to 2.7 μg/kg of dry weight. Moreover, Hu et al. [73] determined the amount of PAEs and phthalate monoesters in soil using ASE as the extraction technique, and the LODs were verified to be in the range of 0.59 to 10.08 ng/g. Wei et al. [74] determined the PAEs levels in samples from soil and vegetables, and the levels varied from 5.42 to 1580 ng/g and from 10.9 to 16,400 ng/g dry weight, respectively. In addition, for surface waters, Liu et al. [75] studied the impact of microplastics and levels of PAEs in surface seawater by solid-phase extraction (SPE) followed by GC-MS, and the concentrations obtained varied from 129.96 ng/L to 921.22 ng/L, while recovery ranged from 84 to 101%. Nagorka and Koschorreck [76] investigated phthalates in suspended particulate matter (SPM) samples from 2000 until 2017. The LODs obtained varied from 0.33 to 43 ng/g with good recoveries rates. Regarding water samples and sediments, Chen and collaborators [68] using UAE coupled with GC-MS, found that the levels varied from 2.65–39.31 μg/L in water, 1.97–34.10 μg/g in SPM, and 0.93–34.70 μg/g in sediments.

### 3.2. Foods

Food contaminants can also occur with the migration of PAEs from packaging to food. In that sense, Arfaeinia et al. [77] investigated the levels of these target compounds in acidic juices. The results showed that DEHP and DnBP were the major compounds identified with the median values of 8.1 and 6.8 µg/L, 10.5 and 7.2 µg/L, and 9.8 and 6.7 µg/ L, in lemon juice, vinegar, and verjuice, respectively. In addition, the results showed that the migration level is higher in PET containers than in glass containers, which indicates that the migration from the wall of the plastic containers to its contents was accelerated at high storage temperatures [77]. On the other hand, the presence of PAEs in glass containers could result from the other processing steps (e.g., storage tanks, filtration steps, and cap-sealing). Regarding baby foods, several authors have investigated many PAEs. Notardonato et al. [78] used an ultrasound-vortex-assisted liquid-liquid microextraction (UVA-DLLME) to extract the amount of PAEs and pesticides in baby food, and DEP, DBP, and DEHP were quantified in almost all the samples at levels ranging between 1 and 40 ng/g. In addition, Socas-Rodríguez et al. [79] determined the levels of 14 PAEs using the quick, easy, cheap, effective, rugged, and safe (QuEChERS) combined with gas chromatography-tandem mass spectrometry (GC-MS/MS). BBP, bis(2-n-butoxyethyl) phthalate (DBEP), DEHA, DEP, and diisodecyl phthalate (DiDP) were found in abundance in the samples particularly DEHA, with concentrations in the range from 0.50 to 8.71 μg/kg, while DPP was only found in plastic-packed products. Furthermore, Pang et al. [80] used the magnetic solid-phase extraction(MSPE) to extract 15 PAEs from beverages, and from these 8 PAEs were detected milk-containing beverages, including dimethyl phthalate (DMP), DEP, bis(4-methyl-2-pentyl) phthalate (BMPP), di-n-amyl phthalate (DPP), dihexyl phthalate (DHXP), BBP, dicyclohexyl phthalate (DCHP), and DnOP. The DEHP concentration in the fresh-made milk tea was 1.69 μg/L. Concerning beverages in plastic containers, Notardonato et al. [61] analyzed the migration of PAEs from plastic containers to beverages using a solvent-based dispersive liquid–liquid microextraction (SB-DLLME) combined with GC-MS. After the release simulation, DiBP, DBP, DHEP, and DnOP were found at very low concentrations (below 1.2 ng/mL) in two water samples from (sport) bottles. Huang et al. [81] also studied PAEs from bottled waters using a hollow fiber-SPME (HF-SPME). After the characterization of fibers, they were applied to the analysis of real samples, with values ranging from 2.42 to 185.95 µg/L for DEP and di(methoxyethyl) phthalate (DMEP), respectively. Additionally, Abtahi et al. [59] also analyzed bottled and tap waters for PAEs content, and the average levels of DEHP, BBP, DBP, DEP, DMP, and DnOP were found to be 0.46 μg/L in surface waters, 0.10 μg/L in groundwaters, 0.17 μg/L in surface waters, 0.18 μg/L in bottled water, 0.52 μg/L in bottled water, and 0.01 μg/L in groundwaters, respectively. Panio and collaborators [82] compared two extraction procedures, namely direct immersion SPME and ultrasonic-assisted solvent extraction (UASE), to determine the levels of PAEs in fish fillets. Using UASE, the values of PAEs ranged from 4.3 to 62.2 µg/kg, and for SPME ranged from 1.3 to 37.4 µg/kg. In addition, the study revealed that SPME provided better control of background contamination than UASE. Ibarra et al. [70] studied the amount of PAEs migration from plastic containers to several classes of foodstuff and beverages using purge and trap coupled to GC–MS. The data showed that migration occurs to a larger extent in tenax than in isooctane. Li et al. [83] also used vortex-assisted liquid–liquid microextraction (VALLME) to analyze PAEs in several food-contacted plastics, and their values ranged from 0.92 to 5.67 µg/g. In addition, Perestrelo et al. [60] used the SPME extraction procedure to analyze food-contacted plastics in which the amounts detected ranged from 1.0 to 2.8 µg/L for BBP and DOP, respectively. Diamantidou and collaborators [84] determined the levels of PAEs in 45 samples from Greek grape marc spirits using the ultra-high-performance liquid chromatography-tandem mass spectrometry (UHPLC-MS/MS) method. The amounts in samples ranged from 1.25 to 113,220 µg/L for dipentyl phthalate (DPeP) and DEHP, respectively. On the other hand, Otoukesh et al. [85] used a graphene oxide/layered double hydroxides@sulfonated polyaniline (GO/LDHs@SPAN) to analyze four PAEs in drinking water and distilled herbal beverages. The data obtained showed that GO/LDHs@SPAN is more efficient than the SPE in extracting PAEs from drinking water and distilled herbal beverages. Notardonato et al. [86] analyzed honey samples to determine the levels of plastic residues by dispersive liquid–liquid microextraction (DLLME) and GC-MS. The lowest concentration was obtained for DnOP with 5.1 ng/g, while the highest was for bisphenol A (BP-A) with 996.8 ng/g. Using honey samples, Notardonato et al. [87] also developed and validated a UVA-DLLME combined with GC-MS to determine PAEs in six honey samples. The highest amount was obtained for DEP with 5.05 µg/g while the lowest for di-isobutyl phthalate (DiBP) with 0.01 µg/g. Dobaradaran et al. [88] analyzed bottled milk samples to determine the levels of PAEs. The study allowed the quantification of five PAEs, which exhibited the highest amounts for DnOP (1261.10 ng/L), while for DMP (2.66 ng/L), lower amounts were obtained. Korkmaz and collaborators [89] also analyzed milk products (36 yogurt and 24 ayran samples) for the presence of PAEs. The lowest and the highest amounts of DBP, DEHP, and BBP in yogurt samples were in the range of 6–229 µg/kg, 24–122 µg/kg, 22–63 µg/kg, respectively. Furthermore, the lowest and highest PAEs amounts in ayran samples were 38–59 µg/kg for DBP and 26–81 µg/kg for DEHP. Concerning olive oil, Pereira et al. [90] analyzed olive oil samples from the European market. All samples analyzed had an average concentration of 1.31 and 1.52 mg/kg for DEHP and with the highest concentration of 7.52 and 6.29 mg/kg for DiNP, respectively. Moreover, Kiralan et al. [91] analyzed different types of olive oils for PAEs levels, and DEHP was the abundant PAEs in all olive oil samples ranging from below the LOQ (0.23 mg/kg) to 602 mg/kg. In all analyzed samples, the levels of DiNP and diisodecyl phthalate (DiDP) were lower than their LOQ. In addition, Perestrelo et al. [69] evaluated the occurrence of PAEs in 20 Portuguese wines by SPME and GC-MS. The values obtained ranged from 0.71 to 23.2 µg/L for DBP. The results also indicated that the PAE concentration depends on the wine quality as well as the aging process, as the choice of the raw material is a critical condition. Aghaziarati at al. [92] developed an electrodeposited terephthalic acid-layered double hydroxide (Cu-Cr) nanosheet coating for the extraction of PAEs from alcoholic beverages. The results confirmed the presence of DMP, DBP, diamyl phthalate (DAP), DEHP in alcoholic beverages. Mirzajani et al. [93] developed and characterize a metal-organic framework-deep eutectic solvents/molecularly imprinted polymers (MOF-DES/MIPs) for the detection of PAEs in yogurt, water and soybean oil samples. Upon validation, the levels of PAEs were determined and ranged from 0.05 to 0.18 µg/L, while recoveries were between 96% and 100%.

## 4. Analytical Approaches

### 4.1. Sample Preparation and Extraction Techniques

Special attention should be given to the sample preparation step as the sample can certainly be contaminated with laboratory material such as solvents (e.g., HEX and EtAc), sorbents (e.g., Florisil and silica gel), plastic consumables (e.g., pipet tips and SPE cartridges), glassware, laboratory air, fibers, stir bar, among others [11,118]. This cross-contamination results in an overestimated contamination levels and/or false positives. Consequently, to overcome the contamination problems, the step involved in the sample preparation, as well as solvent amounts, glassware, extraction time, and exposure of the sample to air, should be minimized [118]. Some studies have been performed to reduce the contamination level in laboratory material [11,119,120,121,122]. Reid et al. [119] performed a screening of common laboratory equipment and components, and the results indicated that plastic syringes, pipette tips produced maximum leaching of 0.36 µg/cm^2^ of DEHP and 0.86 µg/cm^2^ of DiNP, plastic filter holders releases maximum leaching of 2.49 µg /cm^2^ of DBP from polytetrafluoroethylene (PTFE) and Parafilm^®^ leached levels up to 0.50 µg/ cm^2^ of DEHP. To reduce the high levels of PAEs in these materials, a heat or high-temperature process was applied since there is no covalent bond between the PAEs and plastics. Tienpont et al. [122] verified that polypropylene SPE cartridges contained 3 to 14 ng of DiBP, DBP, and DEHP, and in order to reduce this contamination, advised washing the SPE cartridges with an organic solvent. Guo et al. [11] measured PAEs concentration in alumina (100–200 mesh), Florisil (60–100 mesh), and anhydrous sodium sulfate (Na_2_SO_4_), and DiBP and DEHP were found at trace concentration (ng/g) in alumina and Na_2_SO_4_. These authors also measured the PAEs concentration in the commercial solvents (e.g., HEX, acetone (ACET), DCM, and acetonitrile (MeCN)), being DEP (0.01 to 0.03 ng/mL), DiBP (0.002 to 0.21 ng/mL), DBP (0.01 to 0.80 ng/mL), di-n-hexyl phthalate (DnHP, 0.002 to 0.41 ng/mL), benzylbutyl phthalate (BzBP, 0.02 to 0.07 ng/mL), and DEHP (0.28 to 6.39 ng/mL) found in all solvents. The presence of these PAEs in solvents can be reduced by redistillation and/or by SPE [11,118]. Nevertheless, redistillation is not efficient since it can introduce solvents from other sources of contamination (e.g., exposure solvent to laboratory air, requires deactivated alumina), being SPE the most suitable efficient process to remove phthalates from solvents [11,123]. The phthalate contamination in HEX was reduced to 99.8% using 3% of deactivated alumina, but DBP and DEHP were detected at 0.1 ng/mL after purification with activated alumina [123]. Nevertheless, the SPE technique is more suitable for apolar solvents (e.g., pentane and HEX), which will extract PAEs from alumina rather than the phthalates being adsorbed from the solvents [11]. After that, the solvent bottles should be capped to avoid interaction with the surrounding air [118]. Regarding glassware, according to the Fernández-González et al. [121], all material used during sampling and sample preparation should be glass-made and should be washed soaking the material in an alkaline solution for 48 h, rinse with purified water, and then washed gently with methanol (super purity grade). Finally, the glassware should be calcined at 450 °C overnight. In summary, during sample preparation, plastic materials should be avoided, the laboratory material (e.g., solvents and glassware) should not be exposed to air, the glassware should be selected carefully, and the time and the solvent amount involved during sample preparation should be minimized [11,118].

### 4.2. Extraction Techniques

Efficient pre-concentration and clean-up procedures are necessary to guarantee the quality of the analytical methods, due to the predictable low concentration of these target analytes in samples, as well as the sample complexity [118]. Table 2 summarizes the extraction techniques adopted in the last three years to analyze PAEs in environmental and food matrices. It is possible to observe that the most common extraction techniques used to extract phthalates from environmental and food samples are LLE [62,63,64,71,74,76,77,89,91,94,95,96,99,104,106,109,110,112], SPME [60,69,81,82,92,93,115], QuEChERS-dSPE [79,88,102], SPE [68,75], and soxhlet [100,101,114].

In the LLE procedure, the sample is put in contact with a solvent with a high affinity to PAEs, followed by phase-separation caused by solvent properties, centrifugation, and removal of moisture by treatment with Na_2_SO_4_ [11]. In general, no clean-up procedure is necessary. The most common solvents used in LLE are DCM [62,64,66,99], ACET [94], MeCN [90,91,106], and solvents mixes (HEX:ACET, HEX:EtAc, DCM:ACET) [63,71,74,76,77,95,96]. Despite the high extraction efficiency of LLE to extract PAEs from different samples, which results in recovery rates of 70% to 120%, lower LODs and LOQs (Table 2), the up-to-date tendencies related to extraction procedures are founded on the principles of green chemistry, which include low solvent volumes, simplicity, and quickness. In this sense, compared to LLE, dispersive liquid-liquid microextraction (DLLME) is simpler, fast, and environmentally friendly since it requires few µL of solvent volume. DLLME comprises the formation of a cloudy solution endorsed by the rapid injection of a mixture of extractive and dispersive solvents to an aqueous sample with a great contact surface. The droplets formed and dispersed through the aqueous sample are collected by centrifugation, promoting high yields and enrichment factors [124,125]. DLLME using HEX as solvent was used to evaluate the migration of PAEs from different beverage and food plastic containers [61,105], as well as to determine 6 PAEs in waters [117]. The data obtained in these studies confirm the potentiality of DLLME in PAEs extraction since good recoveries (76 to 104%), accuracy (RSD < 10%), low LODs (1.0 to 19 ng/mL), and LOQs (2.1 to 48 ng/mL) were attained. Ultrasound-vortex-assisted DLLME (UVA-DLLME) has been proposed by Notardonato et al. [78], as a modified DLLME with no dispersant solvent, in order to determine 19 organophosphorus pesticides and 6 PAEs in baby food. This method involved analyte extraction using 250 µL of heptane followed by the addition of sodium chloride (NaCl) to break the microemulsion. The results obtained showed that UVA-DLLME is sensitive and more reliable with lower LODs (<4.4 ng/g), LOQs (<7.5 ng/g), high recoveries (91% to 110%), and accuracy (RSD < 10%). Recently, Notardonato et al. [86,87] used UVA-DLLME to determine PAEs and BP-A in honey samples and to evidence the presence of plasticizer residues in nectar honey samples. The advantage of these studies related to the previous one was the reduction in solvent volume, namely 75 µL of heptane [86] and 150 µL of toluene [87]. For both studies, good recoveries (71%–117%), accuracy (RSD < 10%), low LODs (<13 ng/g), and LOQs (<22 ng/g) were achieved, which demonstrate the efficiency of this technique in PAEs extraction. On the other hand, Li et al. [83] used deep eutectic solvents (DESs, 80 µL) as an extraction solvent in a vortex-assisted liquid-liquid microextraction (VALLME) for the extraction and pre-concentration of four PAEs in water and in food-contacted plastics. The LODs and LOQs obtained were lower than 1 and 5 µg/L respectively, and good recoveries (86%–103%) and accuracy (<6%) were obtained, which support that VALLME is a simple, sensitive, fast, efficient, and low-cost extraction technique for the determination of PAEs from food contacting plastics.

Other extraction techniques used in the determination of PAEs are Soxhlet and SPE. Nevertheless, these extraction techniques are time-consuming, require solvents, and extensive sample handling (Table 3), which induces phthalates contamination and are not environmentally friendly [118,121]. In addition to the traditional SPE, magnetic solid-phase extraction (MSPE) using a magnetic covalent organic framework (COF) [80] and a core-shell nanostructured magnetic Ti-silica (Mag@MCM-41/TiO_2_) [116] as adsorbents have been used to determine phthalates in bottle waters.

In contrast to LLE, soxhlet, and SPE, SPME comprises sampling, extraction, purification, concentration, and injection into a single procedure. This extraction procedure is solvent-free, does not need previous sample preparation, and consequently, the risk of cross-contamination from solvents, samples, and glassware was reduced [121]. SPME fibers can be directly immersed (DI) and/or placed in the headspace (HS) of the sample. In HS-SPME, the analytes are released from the gas phase equilibrated with the sample, and in this mode, the fiber is protected from aggressive effects produced by high molecular-weight compounds existing eventually in the sample [121]. However, the main disadvantages of SPME are the price of the fibers and the time to achieve the equilibrium between sample and target analytes (Table 3), which can contribute to inexact quantities [11]. Perestrelo et al. [60,69] used HS-SPME mode to determine four PAEs in table and fortified wines and to assess the occurrence of PAEs in plastic materials used in food packaging. In both studies, using suitable method performance characteristics, recovery (80%–108%), precision (RSD < 13%), and LODs (0.03–0.11 μg/L) and LOQs (0.09–0.36 μg/L) were obtained, which indicate the sensitivity and efficiency of HS-SPME in the PAEs determination. Huang et al. [81] determined 10 PAEs in bottle waters using SPME with polysulfone hollow fiber (HF-SPME). This extraction technique allowed to obtain recovery values in the range of 87%–118%, low LODs (0.001–0.130 µg/L), and accuracy with RSD lower than 10%. The data suggested that HF-SPME is simple, environmentally friendly, and accurate for the determination of phthalates in bottled waters. Mirzajani et al. [93] fabricated, for the first time, monolithic and hollow fiber using a metal–organic framework/deep eutectic solvents/molecularly imprinted polymers (MOF-DES/MIPs) and used for microextraction of PAEs under hollow-fiber liquid membrane-protected solid-phase microextraction (HFLMP-SPME) from yogurt, water, and edible oils. Satisfactory method performance characteristics in terms of recovery, LODs, LOQS, and accuracy were achieved under optimal conditions. Moreover, comparing this study with that of Huang et al. [81], lower LODs (0.01–0.03 µg/L) and LOQs (0.03–0.12 µg/L) were attained, which demonstrated the high sensibility of the HFLMP-SPME compared to HF-SPME. On the other hand, Aghaziarati et al. [92] introduced an electrodeposited terephthalic acid-layered double hydroxide (Cu-Cr) nanosheet coating for in-tube SPME of PAEs in whiskeys. The data obtained in terms of recovery, LODs, LOQs, precision, and accuracy demonstrated the potentiality of IT-SPME on the determination of phthalates.

QuEChERS-dSPE is an extraction technique that comprises two stages, namely extraction and clean-up. The extraction relies on the partitioning via salting-out extraction where an equilibrium between an aqueous and an organic layer (e.g., MeCN) was endorsed, while the clean-up by dSPE used various mixtures of porous sorbents and salts to eliminate matrix interfering compounds [126]. QuEChERS-dSPE has been used to determine PAEs in baby foods [79], beverages [102], and milk [88], and satisfactory method performance characteristics were achieved in these studies, demonstrating the sensitivity of this extraction technique. Nevertheless, special attention should be given to the study performed by Dobaradaran et al. [88] that proposed a novel adsorbent resulting from a combination of multi-walled carbon nanotubes (MWCNT) and iron oxide (Fe_3_O_4_) nanoparticles to extract 10 PAEs from milk. The data obtained, with recoveries ranging from 82% to 112%, and LODs and LOQs lower than 19 and 63 ng/L for all target analytes, supported the successful application of this modified QuEChERS-dSPE approach.

### 4.3. Analytical Approaches

The selection of the most suitable analytical approaches to separate, detect and identify a class of target compounds depends essentially on their physic-chemical properties and the sensitivity requested. As can be observed in Table 2, the most common analytical approaches used for PAEs determination were gas chromatography (GC) and liquid chromatography (LC) combined with mass spectrometry (MS).

#### 4.3.1. Gas Chromatography

Gas chromatography is the most used analytical approach for PAEs determination since these target analytes present low molecular weight (e.g., DMP, DEP, DBP, and DEHP), relatively low polarity, and thermally stable and appropriately volatility [118]. In the case of phthalates with high molecular weight, a derivatization step is required to convert phthalates to their volatiles through the methylation (-COOCH_3_) or silylation (-COOSiR_3_) process of the carboxylic acid group [120]. Nevertheless, excess derivatization agents or byproducts should be removed prior to GC analysis to avoid the deterioration of the stationary phase (column) [120]. Generally, the PAEs separation is carried out using apolar fused-silica capillary columns coated with 5% phenyl and 95% dimethylpolysiloxane, under temperature programs starting from 60 °C up to 330 °C, in total run time analysis ranging from 20 to 51 min [66,67,69,79,85,86,91,100,103]. Nonetheless, the PAEs have also been determined using apolar fused-silica capillary columns coated with 5%-phenyl(1%-vinyl)-methylpolysiloxane (SE-54) [61,78,87]. Conventionally, flame ionization detector (FID) was applied to determine PAEs in sediments [65,97], waters [59,115,117] and foods [93], but FID has been replaced by MS detector due to its specificity and high sensitivity [60,63,66,67,68,69,75,87,94,105,108,114,125]. In the case of GC-MS, it is crucial to use an internal standard (IS) to promote a more accurate quantification of phthalates in a diversity of samples. The isotopically-labeled phthalates as IS [90,100,127], such as d4-DBP, d4-DEHP, and d4-DnOP, are the most suitable to correct errors caused by matrix effects and to correct probable dissimilarities occurring in the analyses, even though non-deuterated compounds like as 2,6-di-ter-butyl-4-methyl phenol (BHT) [91], bromopropylate [78] and anthracene [61] have also been used with good results. Regarding ionization techniques, electron ionization (EI) is the most suitable for the determination of PAEs by GC-MS, being reproducible and not suffering from ion suppression effects [60,61,69,91,100,103,112]. Furthermore, gas chromatography-tandem mass spectrometry (GC-MS/MS) [71,79,80,90,99,102,127] and flash evaporation GC-FID [81] have also been explored as an alternative to determine PAEs in a diversity of samples with excellent results. The tandem MS spectrometry compared to MS demonstrated more sensitivity, mass accuracy, and resolution. Concerning to the analyzer, single quadrupole [60,69,91,100,112], ion trap [61,78] and triple quadrupole (QqQ) [79,102,127] using full scan [60,61,69,103], single ion monitoring (SIM) [90,91,100,112] and multiple reaction monitoring (MRM) [79,102,127] are the most commonly used. The recovery, accuracy, LODs, and LOQs could change based on the extraction technique applied and analytical approaches (Table 2).

In summary, the main advantages of GC-MS are high sensitivity (low LODs), especially by splitless injection, high reproducibility of the generated mass spectra by EI, low cost, ease of operation, requires less maintenance, and the identification of compounds is easier due to the available spectra libraries (e.g., NIST).

#### 4.3.2. Liquid Chromatography

GC is possibly the most used analytical platform in PAEs analysis. Liquid chromatography (LC) appears as a suitable alternative due to its potentiality in the analysis of thermally unstable and non-volatiles compounds providing a high selectivity [125]. High-performance liquid chromatography (HPLC) using C18 analytical columns with an internal diameter (ID) of 4.6 mm running either in isocratic and gradient elution with ultraviolet (UV) detector have been widely used in the measurement of phthalates in meat and water samples [110,116]. Although UV detectors have been demonstrated to be suitable for quantification of PAEs, they do not provide detailed structural information of known target compounds, being this its main drawback. Liquid chromatography coupled with tandem mass spectrometry (LC-MS/MS) [76,82,89] has been explored as an alternative to HPLC-UV, HPLC-DAD since it has shown higher sensitivity, selectivity, resolution, and effectiveness. Moreover, MS detectors are a potent instrument to identify and check molecular structures of unidentified compounds and qualitative analysis. Recently, ultra-performance liquid chromatography coupled with tandem mass spectrometry (UPLC-MS/MS) compared to LC-MS/MS have the potentiality to separate the PAEs in a shorter run time (14 to 16 min) and use smaller particle LC columns (2.1 µm), which allows obtaining narrower peaks and high sample throughput [73,84,101,108]. Electrospray ionization (ESI) interface, triple quadrupole (QqQ), and/or quadrupole time of flight (TOF) mass analyzer and MRM modes are the most common combination used in LC-MS for quantification of PAEs [73,84,101,108]. Diamantidou et al. [84] have proposed a direct UPLC-MS/MS for analysis of 12 PAEs in grape marc spirits of Greek origin. The PAEs were separated using a U-VDSpher PUR 100 C18-E (100 mm × 2.0 mm, 1.8 μm) column by gradient elution. Satisfactory method performance characteristics in terms of recovery (90%–111%), accuracy (RSD < 13%), LODs (0.3–0.33 μg/L), and LOQs (1.0–100 μg/L) were obtained, which indicate the sensitivity and efficiency of direct UPLC-MS/MS in the PAEs determination. Perhaps MS/MS continues to be the method of choice; liquid chromatography-high resolution mass spectrometry (LC-HRMS) has been recently used for the determination of PAEs and their metabolites in seafood species [33,111] and to dust samples from different indoor environments [128]. HRMS coupled to LC for PAEs determination allows better selectivity and sensitivity compared to the low resolution of MS due to accurate masses. Moreover, HRMS provides the possibility of a selection of a very narrow mass window, consequently reducing the chemical background.

In summary, LC-MS offers several benefits in comparison to GC-MS, such as being faster, not requiring derivatization, minimal sample preparation, and facilitating the identification and quantification of a greater diversity of compounds (Table 3). Moreover, contrarily to GC, the LC does not require sample volatilization, which circumvents complications related to the chemical degradation and formation of new products under high temperatures.

## 5. Conclusions

The wide occurrence of phthalates, mainly PAEs, in many products has contributed to the rising concerns about their effects on human health. Nevertheless, the health impacts of PAEs exposure are not completely elucidated. This fact highlights the need for the development of sample preparation and analytical approaches with the purpose of quantifying these target compounds with more accuracy. The evolution of sample preparation has been focused on quickness, simplicity, automatic, low sample handling, low solvent volume, use of green extractant with the aim to reduce the risk of cross-contamination from solvents, samples, and glassware, and also environmentally friendly. The most common extraction techniques used for the measurement of PAEs are LLE and SPME. Regardless, SPME compared to LLE presents several advantages such as being solvent-free, easy to operate, and comprising sampling, extraction, purification, concentration, and injection into a single procedure. Regarding analytical approaches, GC coupled with MS is the most used for the quantification of PAEs, as a result of their well-known volatility. Satisfactory figures of merit in terms of recoveries, accuracy, LODs, and LOQs, was obtained to demonstrate the success of GC-MS in PAEs determination. Despite this fact, a direct UHLC-MS/MS has also been used in PAEs determination with excellent results.

The determination of PAEs in samples represents an inspiring task, not only because of the low concentration of these target compounds but also due to the complexity of the sample and the potential risk of cross-contamination during all steps of the analysis. This problem can be minimized by avoiding extraction techniques that require solvents and only using glassware. However, prior to analysis, all glassware should be submitted to washed soaking in an alkaline solution for 48 h, rinsed with purified solvents, and then calcined at 450 °C overnight. After all, as mentioned in this review, it is expected that miniaturized and automated extraction techniques and high-throughput analytical approaches will continue to be developed to improve the accuracy of PAEs determination.

## Figures and Tables

**Table 1:** The most widely used PAEs and their metabolites [1,2,3,4,5,6].

Phthalate	Chemical Structure	CF ^a^/MW ^b^	Common Uses	Effects	Metabolites ^c^
Butyl benzyl phthalate (BBP)	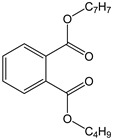	C_19_H_20_O_4_/ 312.4 g/mol (LMW) ^d^	As a plasticizer for vinyl foams, often used as floor tiles. Traffic cones, food conveyor belts, and artificial leather.	Long-term occupational exposure to BBP increase the risk of multiple myeloma, teratogenicity, and reproductive effects.	Mono benzyl phthalate (MBzP)
Di-n-butyl phthalate (DBP)	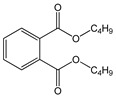	C_16_H_22_O_4_/ 278.3 g/mol (LMW)	As a plasticizer. Most common phthalate added to nail polish.	Suspected teratogenic and endocrine disruptor	Mono-n-butyl phthalate (MBP); Mono-isobutyl phthalate (MiBP)
Di-(2-ethylhexyl) phthalate (DEHP)	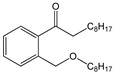	C_24_H_38_O_4_/ 390.6 g/mol (HMW) ^e^	As plasticizers in medical devices, such as intravenous tubing and bags.	Endocrine disruption in males, through its action as an androgen antagonist. Associated with lower levels of reproductive function in adolescent males.	Mono-(2-ethylhexyl) phthalate (MEHP)
Diethyl phthalate (DEP)	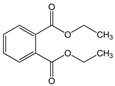	C_12_H_14_O_4_/ 222.2 g/mol (LMW)	Personal care products to enhance fragrances.	Repeated administration of DEP results in loss of germ cell populations in the testis.	Monoethyl phthalate (MEP)
Di-isodecyl phthalate (DiDP)	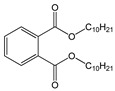	C_28_H_46_O_4_/ 446.7 g/mol (HMW)	Production of plastic and plastic coating.	Reproductive toxicity.	-
Di-isononyl phthalate (DiNP)	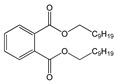	C_26_H_42_O_4_/ 418.6 g/mol (HMW)	Plasticizer. Added as a softener in the manufacture of toys and childcare products.	High concentrations of DiNP in zebrafish disrupt the endocannabinoid system (ECS) and affect reproduction. Upregulates orexigenic signals and causes hepatosteatosis together with deregulation of the peripheral ECS and lipid metabolism.	-

^a^ CF: chemical formula; ^b^ MW: Molecular weigth; ^c^ Major metabolite; ^d^ LMW: lower-molecular weight; ^e^ HMW: higher-molecular weight.

**Table 2:** Analytical approaches used for determination of phthalates esters (PAEs) in environmental and food samples.

Target Analytes	Matrices (Amount)	Extraction Technique (Conditions)	Analytical Tool/Column	Method Performance		Ref.
Environmental						
DMP, DEP, DBP, and DEHP	Mine tailings (5 g)	ASE (2 × DCM)	GC-MS/HP-5MS (30 m × 0.25 mm i.d. × 0.25 µm)	LOD (µg/kg)	1.2–2	[67]
				LOQ (µg/kg)	3.0–4.6	
				RSD (%)	<7	
				Rec. (%)	71–115	
DMP, DEP, DiPrP, DnPrP, DiBP, DBP, DPP, DiHP, BBP, DCHP, DPhP, DEHP, DOP, and DDP	Seawater (2 L)	LLE (2 × 40 mL DCM)	GC-MS/HP-5MS (30 m × 0.25 mm i.d. × 0.25 µm)	LOD (ng/mL)	0.07–0.32	[64]
				LOQ	-	
				RSD (%)	<10	
				Rec. (%)	93–97	
DMP, DEP, DiPrP, DBP, DiBP, BBP, DPhP, DCHP, DHepP, and DEHP	Seawater (500 mL), Sediments (5 g), Seagrass (0.2 g), and Fish (0.2 g)	LLE (30 mL HEX:ACET, 1:1 *v*/*v*), SPE (5 g Floridil, and 60 mL Et2O:HEX, 1:1 *v*/*v*)	GC-MS/SPB-5MS (30 m × 0.25 mm i.d. × 0.25 µm)	LOD (ng/Kg)	5–763	[63]
				LOQ	-	
				RSD (%)	<10	
				Rec. (%)	79–110	
DMP, DEP, DAIP, DiPrP, DnPrP, DiBP, DnBP, DnPeP, BBzP, DCHP, DnHxP, DiHpP, DEHP, DnOP, DiNP, and DiDP	Sediments (5 g)	LLE (3 × DCM), SPE (clean-up, EtAc)	GC-MS/DB-5MS (-)	LOD	-	[62]
				LOQ (ng/g)	0.002–3.92	
				RSD (%)	-	
				Rec. (%)	74–98	
DMP, DEP, DiBP, DBP, BMPP, DMEP, DNPP, DEEP, DNHP, BBP, DEHP, DBEP, DCHP, DnOP, and DNP	Sediments (2 g)	LLE (10 mL HEX:EtAc, 1:1 *v*/*v*)	GC-MS/MS/HP-35MS (30 m × 0.25 mm i.d. × 0.25 μm)	LOD (ng/mL)	0.14–0.88	[71]
				LOQ	-	
				RSD (%)	<15	
				Rec. (%)	71–102	
DBP, BBP, DEHP, DnOP, DiNP, and DiDP	Sediments (5.0 g)	LLE (2 × 10 mL ACET:HEX)	GC-MS/HP-5MS (30 m × 0.25 mm i.d. × 0.25 µm)	LOD (ng/mL)	0.12–1.04	[94]
				LOQ (ng/mL)	1.78–2.98	
				RSD (%)	<9	
				Rec. (%)	81–105	
DMP, DEP, DBP, BBP, DEHP, and DOP	Sediments (2 g)	LLE (DCM:ACET, 7:3 *v*/*v*)	GC-MS/HP-5MS (30 m × 0.25 mm i.d. × 0.25 µm)	LOD (µg/L)	1.25–9.43	[95]
				LOQ (µg/L)	4.17–31.4	
				RSD (%)	-	
				Rec. (%)	72–99	
DMP, DEP, DiBP, DBP, DMEP, DNPP, DeoEP, DNHP, DBEP, BBzP, DMPP, DEHP, DCHO, Dnop, and DnNP	Sediments (5 g) and plants (5 g)	LLE (2 × HEX:ACET, 1:1 *v*/*v*) clean-up SPE (500 mg Florisil, ACET:HEX 1:4 *v*/*v*)	GC-MS/SHR5XLB (30 m × 0.25 mm i.d. × 0.25 µm)	LOD (ppb)	-	[96]
				LOQ (ppb)	2.8–21.2	
				RSD (%)	-	
				Rec. (%)	79–137	
DBP	Sediments (2 g)	UAE (3 × 45 mL DCM)	GC-FID/HP-5 (30 m × 0.25 mm i.d. × 0.25 µm)	LOD	-	[97]
				LOQ (ng/g)	-	
				RSD (%)	-	
				Rec. (%)	-	
DMP, DEP, DiBP, DBP, DMEP, BMPP, DEEP, DPP, DnHP, BBP, DBEP, DCHP, DEHP, DPhP, DnOP, and DiNP	Sediments (0.5 g)	UAE (1 × 2 mL DCM)	GC-MS/DB-5 (30 m × 0.25 mm i.d. × 0.25 µm)	LOD (pg/g)	3–5	[98]
				LOQ	-	
				RSD (%)	<10	
				Rec. (%)	84–119	
DMP, DEP, DBP, DEHP, and DnOP	Sediments (3 g)	Microwave (110 °C), clean-up (2 × 5 mL HEX:TOL 4:1 *v*/*v* and 5 mL EtAC)	GC-FID/DB-5 (30 m × 0.32 mm × 0.25 µm)	LOD (µg/g)	0.015	[65]
				LOQ	-	
				RSD (%)	-	
				Rec. (%)	85–103	
DMP, DEP, DBP, BBP, DEHP, and DnOP	Sediments (20 g) and Water (500 mL)	LLE (30 mL DCM)	GC-MS/DB-5MS (30 m × 0.25 mm × 0.25 µm)	LOD (ng/mL)		[66]
				LOQ (ng/mL)	0.60–0.80	
				RSD (%)	<10	
				Rec. (%)	77–110	
DMP, DEP, DAIP, DiPrP, DnPrP, DiBP, DBP, DnPeP, BBzP, DCHP, DnHxP, DiHpP, DEHP, DnOP, DiNP, and DiDP	Sludge (0.1 g)	LLE (10 mL DCM), clean-up (SPE, 8 mL EtAC)	GC-MS/MS/DB-5MS (30 m × 0.25 mm i.d. × 0.25 μm)	LOD (ng/g)	-	[99]
				LOQ (ng/g)	0.093–196	
				RSD (%)	<21	
				Rec. (%)	68–103	
DMEP, DPP, DBP, DCHP, DnOP, DiNP, and DiDP	Soil (1 g)	MSPD (30 mg MOF, 5 mL MeCN)	UHPLC-MS/MS/BEH C18 (50 mm × 2.1 mm i.d. × 1.7 µm)	LOD (µg/kg)	0.042–0.80	[72]
				LOQ (µg/kg)	0.14–2.7	
				RSD (%)	<20	
				Rec. (%)	70–115	
DMP, DEP, DiBP, DBP, DMGP, DEEP, DCHP, DMPP, BBP, DNHP, HEHP, DBEP, DEHP, and DnOP	Soil (1 g)	ASE-in-line clean-up (MeOH 0.01% FA)	UHPLC-MS/MS/BEH Phenyl (100 mm × 2.1 mm i.d. × 1.7 µm)	LOD (ng/g)	0.59–10.08	[73]
				LOQ (ng/g)	0.93–17.20	
				RSD (%)	<15	
				Rec. (%)	69–131	
DMP, DEP, DBP, BBP, DEHP, and DnOP	Soil (5 g) and Vegetables (1 g)	LLE (20 mL HEX:DCM, 1:1 *v*/*v*)	GC-MS/DB-5 MS (30 m × 0.25 mm i.d. × 0.25 μm)	LOD (ng/g)	0.1–0.5	[74]
				LOQ	-	
				RSD (%)	-	
				Rec. (%)	73–105	
DMP, DEP, DBP, BBP, DEHP, and DnOP	Surface water (500 mL)	SPE (2 mL MeOH, 5 mL EtAC)	GC-MS/DB-5MS (30 m × 0.25 mm i.d. × 0.25 μm)	LOD (ng/L)	0.61–2.96	[75]
				LOQ	-	
				RSD (%)	<9	
				Rec. (%)	84–101	
DMP, DEP, DAP, DMEP, BBP, DIBP, DBP, DBEP, DPP, DcHP, DHP, DHpP, DEHP, DiNP, DiDPP, DPHP, and DiUP	SPM (1 g)	LLE (15 mL ACET:DCM:HEX, 20:20:60 *v*/*v*/*v*; 15 mL HEX/ACET 30/70 *v*/*v*)	LC-MS/HSS T3 (75 mm × 2.1 mm i.d. × 1.7 μm)	LOD (ng/g)	0.33–43	[76]
				LOQ (ng/g)	1–130	
				RSD (%)	<20	
				Rec. (%)	91–117	
DMP, DEP, DiBP, DBP, BBP, and DEHP	Water (1L) and SPM (2 L)	Soxhlet (40 mL HEX:ACE, 8:2 *v*/*v*)	GC-MS/DB-5MS (30 m × 0.25 mm i.d. × 0.25 μm)	LOD (µg/g)	0.1–0.5	[100]
				LOQ (µg/g)	-	
				RSD (%)	-	
				Rec. (%)	71–106	
DMP, DEP, DiBP, DBP, DEHP, and DOP	Water (1 mL), SPM (1 g), and Sediments (1 g)	SPE (500 mg C18, 10 mL MeOH/DCM)	GC-MS/DB-5MS (30 m × 0.25 mm i.d. × 0.25 µm)	LOD (ng/L)	0.54–12.36	[68]
				LOQ (ng/L)	-	
				RSD (%)	<11	
				Rec. (%)	81–112	
**Foods**						
DMP, DEP, DBP, BBP, DEHP, and DnOP	Acidic juice (5 mL)	LLE (20 mL ACET:HEX, 1:1 *v*/*v*)	GC-MS (-)	LOD (ng/L)	0.001–0.002	[77]
				LOQ (ng/L)	0.004–0.008	
				RSD (%)	-	
				Rec. (%)	72–111	
DEP, DMP, BBP, DBP, DiBP, DnOP, and DEHP	Animal tissue (1 g), Vegetable powders (5 g), and Water (0.5 L)	Soxhlet (ACET:HEX, 1:1 *v*/*v*) and SPE (15 mL EtAC)	UPLC-TOF-MS/BEH C18 column (100 mm × 2.1 mm i.d. × 1.7 μm)	LOD (ng/mL)	0.03–0.14	[101]
				LOQ (ng/mL)	0.1–0.50	
				RSD (%)	-	
				Rec. (%)	60–120	
DMP, DEP, DBP, iBcEP, BBP, and DEHP	Baby foods (0.1–0.2 g)	UVA-DLLME (250 µL heptane, 0.1 g NaCl)	GC-MS/SE-54 (30 m × 0.25 mm i.d. × 0.25 μm)	LOD (ng/g)	0.4–4.4	[78]
				LOQ (ng/g)	2.3–7.5	
				RSD (%)	<10	
				Rec. (%)	91–110	
BBP, DBEP, DBP, DCHP, DEEP, DEP, DiDP, DiNP, DiPP, DMEP, DMP, DnOP, DnPP, DPP, and DEHA	Baby foods (10 g)	QuEChERS-dSPE (4 g MgSO_4_, 1 g NaCl, 10 mL MeCN) clean-up dSPE (1.2 g MgSO_4_, 200 mg PSA)	GC-MS/MS/HP-5MS (15 m × 0.25 mm i.d. × 0.25 μm)	LOD (µg/kg)		[79]
				LOQ (µg/kg)	0.03–1.11	
				RSD (%)	<19	
				Rec. (%)	70–120	
DMP, DEP, DiBP, DBP, DMEP, BMPP, DEEP, DPP, DHXP, BBP, DBEP, DCHP, DEHP, DPhP, and DnOP	Beverages (30 mL)	MSPE (COF-(TpBD)/Fe_3_O_4_)	GC-MS/MS/Rxi-5MS (30m × 0.25 μm i.d. × 0.5 μm)	LOD (µg /L)	0.005–2.748	[80]
				LOQ (µg /L)	0.018–9.15	
				RSD (%)	<10	
				Rec. (%)	80–120	
DPP, DMEP, DCHP, DnOP, DiNP, DiDP, DiPP, DEEP, DnPP, BBP, DEHA, and DBEP	Beverages (10 mL)	QuEChERS (4 g MgSO_4_, 1 g NaCl, 10 mL MeCN) and clean-up dSPE (1.2 g MgSO_4_, 200 mg PSA)	GC-MS/MS/HP-5MS (15 m × 0.25 mm i.d. × 0.25 μm)	LOD (µg /mL)	-	[102]
				LOQ (µg /mL)	0.034–1.415	
				RSD (%)	<20	
				Rec. (%)	75–120	
DMP, DEP, DiBP, DBP, DEHP, and DOP	Beverages plastic containers (10 mL)	DLLME (40 µL HEX)	GC-MS/SE-54 (30 m × 0.25 mm i.d. × 0.25 μm)	LOD (ng/mL)	0.1–1.2	[61]
				LOQ (ng/mL)	2.1–4.9	
				RSD (%)	<13	
				Rec. (%)	76–102	
DPRP, DEP, DBP, DiBP, DPP, DMEP, BBP, DnHP, DEHP, and DnOP	Bottled water (4 mL)	HF-SPME (PSF fiber)	FE-GC-FID /DB-5 (30 m × 0.25 mm i.d. × 0.25 µm)	LOD (µg/L)	0.001–0.130	[81]
				LOQ	-	
				RSD (%)	<10	
				Rec. (%)	87–118	
DEHP, BBP, DBP, DEP, DMP, and DnOP	Bottled water (2 L) and Tap water (2 L)	MSPE (C18, 3 mL MeOH:DCM, 1:1 *v*/*v*)	GC-FID/CP-Sil 8 CB (30 m × 0.32 mm i.d. × 0.25 µm)	LOD (ng/L)	17–31	[59]
				LOQ	-	
				RSD (%)	<20	
				Rec. (%)	98–102	
DMP, DEP, DPrP, DiBP, DBP, DMEP, BMPP, DEEP, DPP, DHP, BBP, DCHP, DEHP, and DnOP	Brands (5 mL), Rice (0.5 g), Wheat (0.5 g), and Sorghum (0.5 g)	VSLLME (500 µL C_2_Cl_4_, 125 µL Tween-20) QuEChERS-dSPE (0.32 g NaCl, 0.70g MgSO_4_, 2 mL MeCN)	GC-MS/TG-5MS (30m × 0.25 μm × 0.25 μm)	LOD (µg/L)	0.05–2.50	[103]
				LOQ (µg/L)	0.125–5.00	
				RSD (%)	<10	
				Rec. (%)	85–121	
DEHP and DBP	Edible vegetable oil (0.5 g)	LLE (2 × 2 mL MeCN + 100 µL Hex) and clean-up SPE (5 mL MeCN)	GC-MS/Rtx-5MS (30 m × 0.25 mm i.d. × 0.25 μm)	LOD (ng/mL)	-	[104]
				LOQ	-	
				RSD (%)	-	
				Rec. (%)	-	
DMP, DEP, DBP, BBzP, and DEHP	Fish fillets (2 g)	SPME (C18 fibers)/UASE (Acet:HEX 1:1, *v*/*v*)	LC-MS/MS/Accucore C-18 aQ (100 mm × 2.1 mm i.d. × 2.6 mm)	LOD (µg/kg)	0.1–0.5	[82]
				LOQ (µg/kg)	0.3–1.5	
				RSD (%)	<24	
				Rec. (%)	-	
DMP, DEP, DiBP, DBP, DEHP, and DnOP	Food contacted plastics (1 L)	DLLME (200 µL HEX)	GC-MS/TRB-Meta X5 (30 m × 0.25 mm i.d. × 0.25 μm)	LOD (ng/mL)	1.0–8.0	[105]
				LOQ (ng/mL)	5.0–14	
				RSD (%)	<10	
				Rec. (%)	93–104	
DEHP, DEP, DiBP, and DBP	Food contact plastics (2 g)	LLE (20 mL MeCN)	GC-MS/ZB-5MS (30 m × 0.25 mm i.d. × 0.25 μm)	LOD (ng/mL)	1–13.3	[106]
				LOQ (ng/mL)	2.5–36.3	
				RSD (%)	<16	
				Rec. (%)	83–116	
DnPP, DAP, BBP, and DOP	Food contact plastics (0.8 g)	VALLME (80 µL DES)	GC-MS/HP-5MS (30 m × 0.25 mm i.d. × 0.25 µm)	LOD (µg/L)	1	[83]
				LOQ (µg/L)	5	
				RSD (%)	<6	
				Rec. (%)	86–103	
DBP, BBP, BDE, and DOP	Food contact plastics (2 mL)	SPME (0.2 g NaCl, PDMS/DVB)	GC-MS/HP-5 (60 m × 0.25 mm i.d. × 0.25 μm)	LOD (µg/L)	0.03–0.08	[60]
				LOQ (µg/L)	0.10–0.24	
				RSD (%)	<13	
				Rec. (%)	90–111	
DMP, DEP, DBP, BBP, DEHP, and DnOP	Foodstuffs (1 g for solids, 200 mL liquids)	UAE (DCM, 30 min), clean-up with GP-MSE (10 µL DCM, 2 min, 280 °C)	GC-MS/DB-5 (30 m × 0.25 mm i.d. × 0.25 μm)	LOD (ng/g) solid	0.14–0.38	[107]
				LOD (ng/L) liquid	2.1–9.6	
				RSD (%)	<10	
				Rec. (%)	86–103	
DMP, DBP, DEP, DPeP, DPP, DEHP, DiPP, DnOP, DPhP, DiNP, BBP, and DiDP	Grape marc spirit (-)		UHPLC-MS/MS/U-VDSpher PUR 100 C18-E (100 mm × 2.0 mm i.d. × 1.8 µm)	LOD (µg/L)	0.3–33.3	[84]
				LOQ (µg/L)	1.0–100	
				RSD (%)	<10	
				Rec. (%)	82–110	
DMP, DBP, BBP, and DEHP	Herbal beverages (10 mL) and Water (10 mL)	UA-D-SPE (5 mg hybrid nanocomposite)	GC-MS/HP-5MS (30 m × 0.25 mm i.d. × 0.25 µm)	LOD (ng/mL)	0.06–0.3	[85]
				LOQ (ng/mL)	0.20–1.00	
				RSD (%)	<12	
				Rec. (%)	55–113	
DMP, DEP, DiBP, DBP, DEHP, and DNOP	Honey (2.5 g)	UVA-DLLME (75 µL benzene, NaCl 10 g/L)	GC-MS/TRB-5MS (30 m × 0.25 mm i.d. × 0.25 μm)	LOD (ng/g)	3.0–13	[86]
				LOQ (ng/g)	7.0–22	
				RSD (%)	<10	
				Rec. (%)	71–10	
DMP, DEP, DiBP, DBP, DEHP, and DnOP	Honey (2.5 g)	UVA-DLLME (150 µL TOL, and NaCl 10 g/L)	GC-MS/ SE-54 (30 m × 0.25 mm i.d. × 0.25 μm)	LOD (ng/g)	2.0–6.0	[87]
				LOQ (ng/g)	7.0–11	
				RSD (%)	<4	
				Rec. (%)	86–117	
BBP, DAP, DBEP, DCHP, DEEP, DiDP, DiNP, DiPP, DNOP, DNPP, and DPP	Jellies (25 mL) and Apple-based beverages (25 mL)	m-µ-dSPE (40 mg Fe_3_O_4_@PPy, 2 mL MeCN)	UHPLC-MS/MS/BEH C18 (50 mm × 2.0 mm i.d. × 1.7 µm)	LOD (µg/L)	-	[108]
				LOQ (µg/L)	0.15–0.42	
				RSD (%)	<20	
				Rec. (%)	60–114	
DMP, DEP, DBP, DEHP, and DnOP	Milk (10 mL)	QuEChERS-dSPE (0.01 g MWCNT-Fe_3_O_4_ and 0.5 g NaCl, 5 mL MeCN)	GC-MS/HP-5MS (30 m × 0.25 mm i.d. × 0.25 µm)	LOD (ng/L)	1.2–19	[88]
				LOQ (ng/L)	3.3–63	
				RSD (%)	<7	
				Rec. (%)	82–112	
DBP, DEHP, BBP, DiNP, DNOP, and DiDP	Milk products (2 g)	LLE (2mL MeOH, 2 mL HEX, 2 mL TBME)	LC-MS/MS/Zorbax SB-C18 (50 m × 2.1 mm i.d. × 1.8 µm)	LOD (µg/kg)	6.0–9.0	[89]
				LOQ (µg/kg)	20–30	
				RSD (%)	<20	
				Rec. (%)	84–96	
DBP, BBP, DEHP, DiNP, and DiDP	Olive oil (1 g)	LLE (10 mL MeCN)	GC-MS/MS/Rxi-5MS (30 m × 0.25 μm i.d. × 0.25 μm)	LOD (ng/mL)	7–130	[90]
				LOQ	23–420	
				RSD (%)	<4	
				Rec. (%)	90–108	
DBP, BBP, DEHP, DiDP, and DiNP	Olive oil (1 g)	LLE (10 mL MeCN)	GC-MS/HP-5MS (30 m × 0.25 mm i.d. × 0.25 μm)	LOD (mg/kg)	0.06–1.97	[91]
				LOQ (mg/kg)	0.09–2.28	
				RSD (%)	<12	
				Rec. (%)	87–100	
DEHP, BBP, DiDP, DBP, and DiNP	Pork (0.5 g) and Chicken (0.5 g)	LLE (3 mL PENT:MeOH 1:4 *v*/*v*)	LC-MS/MS/BEH C18 (100 m × 2.1 mm i.d. × 1.7 μm)	LOD (ng/g)	-	[109]
				LOQ (ng/g)	40	
				RSD (%)	<10	
				Rec. (%)	96–103	
DBP	Red lettuce (-)	LLE (20 mL DCM)	HPLC-UV/Venusil C18 (250 mm × 4.6 mm i.d. × 5 µm)	LOD	-	[110]
				LOQ	-	
				RSD (%)	-	
				Rec. (%)	-	
MMP, MEP, MBP, MBzP, MEHP, MOP, DMP, DEP, BzBP, DBP, DEHP, and DnOP	Seafood species (1 g)	QuEChERS (4 g MgSO_4_, 1 g NaCl, 0.5 g SCDE, 1 g SCTD, 10 mL MeCN), and clean-up dSPE (200 mg C18)	LC-HRMS/Ascentis Express C18 (100 mm × 2.1 mm i.d. × 2.7 µm)	LOD (ng/g)	1–100	[111]
				LOQ (ng/g)	5–250	
				RSD (%)	<15	
				Rec. (%)	13–79	
MMP, MEP, DMP, MBP, MBzP, DEP, MEHP, MOP, BzBP, DBP, DEHP, and DOP	Seafood species (1 g)	ASE (MeOH, 80 °C, 10 min, 1500 psi), clean-up SPE (200 mg bond elut plexa and 5 mL MeOH)	LC-HRMS/Ascentis Express C18 (100 mm × 2.1 mm i.d. × 2.7 µm)	LOD (µg/L)	0.5–25	[33]
				LOQ (µg/L)	1–50	
				RSD (%)	<25	
				Rec. (%)	6–76	
DMP, DEP, DiBP, DBP, DMEP, BMPP, DEEP, DPP, DHXP, BBP, DBEP, DCHP, DEHP, DPhP, DnOP, and DNP	Suet Oil (1 g)	LLE (2 × 5 mL MeCN (HEX saturared))	GC-MS/CD-5MD (30 m × 0.25 mm i.d. × 0.25 μm)	LOD (ng/mL)	0.10–0.70	[112]
				LOQ	0.33–2.31	
				RSD (%)	<10	
				Rec. (%)	83–106	
BBP, DiBP, DnPP, DnOP, DiNP, and DiDP	Tea (10 mL), Apple juice (10 mL), and Pineapple juice (10 mL)	VA-EDLLME (440 µL DES ChCl:phenol 1:2)	LC-DAD-MS/MS/X-BridgeC18 (100 m × 4.6 mm i.d. × 3.5 µm)	LOD (µg/L)	5.1–17.8	[113]
				LOQ (µg/L)	17.2–59.4	
				RSD (%)	<20	
				Rec. (%)	84–120	
DMP, DBP, BBP, DEHP, DnOP, and DEP	Vegetables (2 g) and soil (10 g)	Soxhlet (220 mL MeOH:ACET, 1:1 *v*/*v*)	GC-MS/DB-5MS (30 m × 0.25 mm i.d. × 0.25 μm)	LOD (µg/kg)	0.032–0.191	[114]
				LOQ	-	
				RSD (%)	<11	
				Rec. (%)	70–120	
DEP, DPP, DAP, DBP, BBP, and DEHP	Water (-)	SPME (OH50%-TPB-COF fiber)	GC-FID/HP-5 (50 m × 0.32 mm i.d. × 0.52 µm)	LOD (µg/L)	0.032–0.451	[115]
				LOQ	-	
				RSD (%)	<10	
				Rec. (%)	79–100	
DEP, DPrP, DiBP, and DCHP	Water (20 mL)	MSPE (20 mg MagC-TA, 500 µL MeCN)	HPLC-UV/InertSustain-C18 (250 m × 4.6 μm i.d. × 5 μm)	LOD (ng/mL)	0.10–0.62	[116]
				LOQ	0.33–2.06	
				RSD (%)	-	
				Rec. (%)	82–118	
DEHP, DBP, DiNP, DiDP, and DEP	Water (10 mL)	DLLME (250 µL Heptane, 1 g NaCl)	GC-FID/TRB-Meta X5 (30 m × 0.25 mm i.d. × 0.25 μm)	LOD (ng/mL)	2.0–19	[117]
				LOQ (ng/mL)	4.0–48	
				RSD (%)	<10	
				Rec. (%)	82–102	
DBP, BBP, BDE, and DOP	Wines (2 mL)	SPME (0.2 g NaCl, PDMS/DVB)	GC-MS/HP-5 (60 m × 0.25 μm i.d. × 0.25 μm)	LOD (µg/L)	0.03–0.11	[69]
				LOQ (µg/L)	0.09–0.36	
				RSD (%)	<13	
				Rec. (%)	80–108	
DMP, DBP, DAP, and DEHP	Whisky (10 mL)	IT-SPME (15 % *w*/*v* NaCl and TPA/LDH)	HPLC-UV/ODS-3 (250 m × 4.6 μm i.d. × 5 μm)	LOD (µg/L)	0.01–0.1	[92]
				LOQ (µg/L)	0.03–0.2	
				RSD (%)	<7	
				Rec. (%)	92–112	
DMP, DEP, DIBP, and DBP	Yogurt (1 g), Water (1 g), and Edible oil (1 g)	HFLMP-SPME (monolithic fiber, 6 µL n-hexane)	GC-FID/BP-5 (25 m × 0.32 mm i.d. × 0.5 µm)	LOD (µg/L)	0.01–0.03	[93]
				LOQ (µg/L)	0.03–0.12	
				RSD (%)	<5	
				Rec. (%)	96–100	

Abbreviations: ASE: Accelerated solvent extraction; ACET: acetone; BBP: butyl benzyl phthalate; BEHP: bis(2-ethylhexyl) phthalate; BMPP: bis(4-methyl-2-pentyl) phthalate; BzBP: benzyl butyl phthalate; C_2_Cl_4_: tetrachloroethylene; DAP: diamyl phthalate; DBEP: bis(2-n-butoxyethyl) phthalate; DBP: dibutyl phthalate; DCHP: dicyclohexyl phthalate; DCM: dichloromethane; DDP: diphenyl phthalate; DEEP: bis(2-ethoxyethyl) phthalate; DEHP: di(2-ethylhexyl) phthalate; DEP: di(2-ethylhexyl) phthalate; DES: deep eutectic solvent; DHXP: dihexyl phthalate; DIBP: di-isobutyl phthalate; DiDP: di-isodecyl phthalate; DiHP: di-isoheptyl phthalate; DiNP: di-isononyl phthalate;DiPrP: di-isopropyl phthalate; DLLME: dispersive liquid-liquid microextraction; DMEP: di(methoxyethyl) phthalate; DMP: dimethyl phthalate; DnOP: di-n-octyl phthalate; DNP: dinonyl phthalate; DnPP: di-n-pentyl phthalate; DnPrP: di-n-propyl phthalate; DOP: dioctyl phthalate; DPhP: diphenyl phthalate; DPP: di-n-amyl phthalate; EPA: environmental protection agency; EtAc: ethyl acetate; Et_2_O: diethyl ether; GC-FID: gas chromatography with flame ionization detection; GC-MS: gas chromatography-mass spectrometry; GC-MS/MS: gas chromatography tandem mass spectrometry; GP-MSE: gas purge microsyringe extraction; HEX: hexane; HF-SPME: hollow fiber-solid phase microextraction; HPLC-UV: liquid chromatography with ultraviolet detection; IT-SPME: in-tube solid-phase microextraction; LC-DAD-MS/MS: liquid chromatography coupled to diode array detection tandem mass spectrometry; LC-HRMS: liquid chromatography-high resolution mass spectrometry; LC-MS/MS: liquid chromatography with tandem mass spectrometry; LLE: liquid-liquid extraction; LOD: limit of detection; LOQ: limit of quantification; m-µ-dSPE- magnetic-micro-dispersive solid phase extraction; MBP: monobutyl phthalate; MBzP: monobenzyl phthalate; MEP: monoethyl phthalate; MEHP: mono(2-ethylhexyl)phthalate; MeCN: acetonitrile; MeOH: methanol; MgSO_4:_ sulphate magnesium; MMP: monomethyl phthalate; MOP: monooctyl phthalate; MSPD: matrix solid phase dispersion; MSPE: magnetic solid phase extraction; NaCl: sodium chloride; PDMS/DVB: polydimethylsiloxane/divinylbenzene; PENT: pentane; PSA: primary secondary amine; QuEChERS-dSPE: quick, easy, cheap, effective, rugged, and safe: dispersive solid phase extraction; RSD: relative standard deviation; SCDE: sodium citrate dibasic sesquihydrate; SCTD: sodium citrate tribasic dihydrate; SPE: solid phase extraction; SPME: solid phase extraction; SPM: suspended particulate matter; TOL: toluene; UA-D-SPE: ultrasound-assisted dispersive-solid phase extraction; UAE: ultrasound assisted extraction; UHPLC-MS/MS: ultra-high performance liquid chromatography-MS/MS; UPLC-TOF-MS: ultra-performance liquid chromatography coupled time-of-flight mass spectrometry; UVA-DLLME: ultrasound vortex assisted dispersive liquid–liquid microextraction; VA-EDLLME: vortex assisted-emulsification dispersive liquid-liquid microextraction; VSLLME: vortex-assisted surfactant-enhanced emulsification liquid–liquid microextraction.

**Table 3:** Advantages and disadvantages of extraction technique and analytical approaches used for determination of phthalates esters (PAEs) in environmental and food samples.

Extraction Technique		Advantages	Disadvantages
LLE		✓ Economical ✓ High extraction efficiency ✓ Simple operation ✓ Suitable for small scale	✓ Large volume of solvent ✓ Low selectivity ✓ Difficult to automate ✓ Time-consuming
DLLME assisted (UVA-DLLME, VSLLME, VALLME, VS-EDLLME)		✓ Economical ✓ High recovery ✓ Low amount of sample ✓ Low extraction time ✓ Low volume of solvent	✓ Low selectivity ✓ Requires centrifugation ✓ Requires the use of three solvents
Soxhlet		✓ Simple operation ✓ Suitable for small scale	✓ Large volume of solvent ✓ Limited extraction efficiency ✓ Time-consuming
ASE		✓ Easy of automatization ✓ High efficiency ✓ Low volume of solvent ✓ Short extraction time	✓ Requires high temperatures (40 to 200 °C) and pressures (1500 psi)
UAE		✓ Economical ✓ Environmentally friendly ✓ High extraction efficiency ✓ Short extraction time ✓ Thermally stable molecules	✓ Decline of power with the time ✓ Lack of uniformity in the distribution of ultrasound energy
Microwave assisted		✓ Environmentally friendly ✓ High recovery ✓ Low volume of solvent ✓ No clean-up ✓ Reduced extraction time	✓ Expensive ✓ Low selectivity ✓ Requires centrifugation/filtration
SPE		✓ Alternative of LLE ✓ Easy automation ✓ Suitable for large scale	✓ Involve many steps ✓ Large volume of solvent ✓ Possibility of low recoveries
MSPE		✓ Environmentally friendly ✓ Limited number of steps ✓ Low amount of sorbent material ✓ Reuse of sorbent material ✓ Short extraction time	✓ Requires vortex/shaker/magnetic stirrer ✓ Selection of suitable sorbent
MSDP		✓ Environmentally friendly ✓ Limited number of steps ✓ Quick ✓ Simple	✓ Requires anhydrous sorbents activated at high temperatures
SPME		✓ Alternative to SPE ✓ Limited number of steps ✓ Low amount of samples ✓ Reuse of the polymeric phase ✓ Short extraction time	✓ Potential contamination of the SPME needle
QuEChERS-dSPE		✓ Economical ✓ Efficient clean-up by dSPE ✓ Limited solvent consumption ✓ Quick ✓ Simple	✓ Reduced precision and accuracy ✓ Reduced sensitivity
**Analytical platforms**			
GC	FID	✓ Economical ✓ High sensitivity ✓ Quick ✓ Wide linear range	✓ No information related to structure ✓ Time consuming
	EI-MS	✓ Volatiles ✓ Economical ✓ High resolution ✓ Information related to structure ✓ Library database ✓ Minimal matrix effect ✓ User friendly	✓ Hard ionization ✓ Impossible analysis of thermally stable molecules ✓ Low response factor consistency ✓ Low-volatility compounds need to be derivatized ✓ Moderate sensitivity
LC	UV	✓ Economical ✓ High sensitivity; ✓ HPLC columns can be reused without repacking or regeneration ✓ Speed of analysis ✓ User friendly	✓ Sensitivity is chromophore dependent ✓ Low specificity at short wavelengths ✓ Identification based on retention time and UV/vis absorbance
	ESI-MS	✓ Derivatization is unnecessary ✓ High sensitivity ✓ Large mass range ✓ Mid- to high chromatographic resolution; ✓ Nonvolatile, polar, and ionic molecules (mid- to high-polarity) ✓ Soft ionization ✓ Speed of analyses ✓ Thermally stable molecules	✓ Expensive ✓ Matrix effect ✓ No universal Library ✓ Limited potential in identification unless the MS/MS is used ✓ De-salting may be required ✓ A few restrictions on LC eluents ✓ Low limit of detection (few pg to 10^−15^ g)
	HRMS	✓ High resolution ✓ Mass accuracy ✓ High selectivity ✓ High specificity	✓ Expensive ✓ Instrument maintenance ✓ Data file storage

Abbreviations: ASE: Accelerated solvent extraction; DLLME: dispersive liquid-liquid microextraction; EI-MS: electron ionization mass spectrometry; ESI-MS: electrospray ionization mass spectrometry; FID: flame ionization detection; GC: gas chromatograph; HRMS: high resolution mass spectrometry; LC: liquid chromatography; LLE: liquid-liquid extraction; MS/MS: tandem mass spectrometry; MSPD: matrix solid phase dispersion; MSPE: magnetic solid phase extraction; QuEChERS-dSPE: quick, easy, cheap, effective, rugged, and safe: dispersive solid phase extraction; SPE: solid phase extraction; SPME: solid phase extraction; UAE: ultrasound assisted extraction; UV: ultraviolet detection; UVA-DLLME: ultrasound vortex assisted dispersive liquid–liquid microextraction; VA-EDLLME: vortex assisted-emulsification dispersive liquid-liquid microextraction; VSLLME: vortex-assisted surfactant-enhanced emulsification liquid–liquid microextraction.

## Supplementary Materials

The following are available online at https://www.mdpi.com/article/10.3390/toxics9070157/s1. List of abbreviations.
