# Supplementary material for: Comprehensive Insight from Phthalates Occurrence: From Health Outcomes to Emerging Analytical Approaches
Source: Toxics. 2021 Jul 1;9(7):157. doi: 10.3390/toxics9070157 (PMC8309855; doi:10.3390/toxics9070157)
Supplement: Supplementary file 1 [file toxics-09-00157-s001.zip › toxics-1245717-supplementary.pdf]

toxics

Review

Comprehensive Insight from Phthalates Occurrence: From Health Outcomes to Emerging Analytical Approaches

Catarina Luís, Manuel Algarra, José S. Câmara and Rosa Perestrelo

List of abbreviations:

ACET—acetone
ASE—Accelerated solvent extraction
BBP—butyl benzyl phthalate
BEHP—bis(2-ethylhexyl) phthalate
BHT—2,6-di-ter-butyl-4-methyl phenol
BMPP—bis(4-methyl-2-pentyl) phthalate
BzBP—benzyl butyl phthalate
C_2Cl_4—tetrachloroethylene
DAP—diamyl phthalate
DBEP—bis(2-n-butoxyethyl) phthalate
DBP—dibutyl phthalate
DCHP—dicyclohexyl phthalate
DCM—dichloromethane
DDP—diphenyl phthalate
DEEP—bis(2-ethoxyethyl) phthalate
DEHP—di(2-ethylhexyl) phthalate
DEP—di(2-ethylhexyl) phthalate
DES—deep eutectic solvent
DHXP—dihexyl phthalate
DIBP—di-isobutyl phthalate
DiDP—di-isodecyl phthalate
DiHP—di-isoheptyl phthalate
DiNP—di-isononyl phthalate
DiPrP—di-isopropyl phthalate
DLLME—dispersive liquid-liquid microextraction
DMEP—di(methoxyethyl) phthalate
DMP—dimethyl phthalate
DnOP—di-n-octyl phthalate
DNP—dinonyl phthalate
DnPP—di-n-pentyl phthalate
DnPrP—di-n-propyl phthalate
DOP—dioctyl phthalate
DPeP—dipentyl phthalate
DPhP—diphenyl phthalate

DPP—di-n-amyl phthalate
EFSA—European Food Safety Agency
EPA—Environmental Protection Agency
Et_2O—diethyl ether
EtAc—ethyl acetate
GC-FID—gas chromatography with flame ionization detection
GC-MS—gas chromatography-mass spectrometry
GC-MS/MS—gas chromatography tandem mass spectrometry
GP-MSE—gas purge microsyringe extraction
HEX—hexane
HF-SPME—hollow fiber-solid phase microextraction
HMW—higher-molecular weight
HPLC-UV—liquid chromatography with ultraviolet detection
IT-SPME—in-tube solid-phase microextraction
LC-DAD-MS/MS—liquid chromatography coupled to diode array detection tandem mass spectrometry;
LC-HRMS—liquid chromatography-high resolution mass spectrometry
LC-MS/MS—liquid chromatography with tandem mass spectrometry
LLE—liquid-liquid extraction
LMW—lower-molecular weight
LOD—limit of detection
LOQ—limit of quantification
m-μ-dSPE- magnetic-micro-dispersive solid phase extraction
MBP—monobutyl phthalate
MBzP—monobenzyl phthalate
MeCN—acetonitrile
MEHP—mono(2-ethylhexyl)phthalate
MeOH—methanol
MEP—monoethyl phthalate
$MgSO_4$—sulphate magnesium
MMP—monomethyl phthalate
MOP—monooctyl phthalate
MRM—multiple reaction monitoring
MSPD—matrix solid phase dispersion
MSPE—magnetic solid phase extraction
NaCl—sodium chloride
PAEs—phthalate esters
PDMS/DVB—polydimethylsiloxane/divinylbenzene
PENT—pentane
PET—polyethylene terephthalate
PSA—primary secondary amine
PTFE—polytetrafluoroethylene
PVC—polyvinyl chloride

QuEChERS-dSPE—quick, easy, cheap, effective, rugged, and safe—dispersive solid phase extraction
RSD—relative standard deviation
SCDE—sodium citrate dibasic sesquihydrate
SCTD—sodium citrate tribasic dihydrate
SPE—solid phase extraction
SPM—suspended particulate matter
SPM—suspended particulate matter
SPME—solid phase extraction
TDI—total daily intakes
TOL—toluene
UA-D-SPE—ultrasound-assisted dispersive-solid phase extraction
UAE—ultrasound assisted extraction
UHPLC-MS/MS—ultra-high performance liquid chromatography-MS/MS
UPLC-TOF-MS—ultra-performance liquid chromatography coupled time-of-flight mass spectrometry
UVA-DLLME—ultrasound vortex assisted dispersive liquid–liquid microextraction
VA-EDLLME—vortex assisted-emulsification dispersive liquid-liquid microextraction
VSLLME—vortex-assisted surfactant-enhanced emulsification liquid–liquid microextraction
